# Structural Refolding and Thermal Stability of Myoglobin in the Presence of Mixture of Crowders: Importance of Various Interactions for Protein Stabilization in Crowded Conditions

**DOI:** 10.3390/molecules26092807

**Published:** 2021-05-10

**Authors:** Zahoor Ahmad Parray, Faizan Ahmad, Md. Imtaiyaz Hassan, Anwar Ahmed, Fahad N. Almajhdi, Ajamaluddin Malik, Tajamul Hussain, Asimul Islam

**Affiliations:** 1 Centre for Interdisciplinary Research in Basic Sciences, Jamia Millia Islamia, New Delhi 110025, India; zaparray@gmail.com (Z.A.P.); faizan.ahmad.jmi@gmail.com (F.A.); mihassan@jmi.ac.in (M.I.H.); 2 Centre of Excellence in Biotechnology Research, College of Science, King Saud University, Riyadh 11451, Saudi Arabia; anahmed@ksu.edu.sa (A.A.); majhdi@ksu.edu.sa (F.N.A.); thussain@ksu.edu.sa (T.H.); 3 Department of Botany and Microbiology, College of Science, King Saud University, Riyadh 11451, Saudi Arabia; 4 Department of Biochemistry, College of Science, King Saud University, Riyadh 11451, Saudi Arabia; amalik@ksu.edu.sa

**Keywords:** cellular crowding, synthetic crowders, myoglobin, protein stability, isothermal titration calorimetry, molecular docking

## Abstract

The intracellular environment is overcrowded with a range of molecules (small and large), all of which influence protein conformation. As a result, understanding how proteins fold and stay functional in such crowded conditions is essential. Several in vitro experiments have looked into the effects of macromolecular crowding on different proteins. However, there are hardly any reports regarding small molecular crowders used alone and in mixtures to observe their effects on the structure and stability of the proteins, which mimics of the cellular conditions. Here we investigate the effect of different mixtures of crowders, ethylene glycol (EG) and its polymer polyethylene glycol (PEG 400 Da) on the structural and thermal stability of myoglobin (Mb). Our results show that monomer (EG) has no significant effect on the structure of Mb, while the polymer disrupts its structure and decreases its stability. Conversely, the additive effect of crowders showed structural refolding of the protein to some extent. Moreover, the calorimetric binding studies of the protein showed very weak interactions with the mixture of crowders. Usually, we can assume that soft interactions induce structural perturbations while exclusion volume effects stabilize the protein structure; therefore, we hypothesize that under in vivo crowded conditions, both phenomena occur and maintain the stability and function of proteins.

## 1. Introduction

Proteins fold and operate under cellular conditions in an environment heavily crowded by DNA, RNA, lipids, macromolecules and metabolites. The fact that such an environment, i.e., the crowding, can heavily influence protein folding, stability and function has been repeatedly shown, but in regard to countless details, we are still in the dark [1,2,3,4,5]. The cell is comprised of heterogeneous mixture of various polymers and macromolecules in definite conditions which influence proteins in different ways, such as during folding processes [6,7,8,9,10,11,12,13]. Proteins are exposed to various macromolecules of various sizes, shapes and concentrations within a living cell, executing their functions [14]. The various in vitro studies of proteins in the presence of natural crowders (DNA, proteins, carbohydrate etc.) and synthetic crowders (Ficoll, dextran and PEGs) put together the information on how proteins interact in such crowded solutions, resulting in their stabilization and/or destabilization [6,7,8,9,10,11,12,13,15]. Such studies impersonate the state of crowding with that of in vivo which is highly crowded [16,17]. Highly crowded conditions of intra and inter-cellular can considerably affect the folding, stability, and biophysical properties of proteins [18,19,20,21,22,23,24]. All cells and probably all organelles appear to have various kinds of structuring proteins, often peripherally associated with or penetrating through a cytoplasmic inner sheet of the phospholipid bilayer, contributing to the overall mechanical strength, shape and function of a cell or an organelle [25]. The research on protein folding intermediates has not yet led to final results but work is in progress. Many ideas about their involvement in molecular mechanisms of biological processes have still to be tested [26]. The sighting of intermediate states [27] provides novel insights towards the importance of structural changes of proteins inside cells, where intermediate states of proteins import-export easily via membranes rather than their native forms [28]. These intermediate structures in the cell influence these mechanisms to address emerging challenges in developing therapeutics and precision medicine [29,30]. To maintain the stability and function of different existing intermediate states (such as molten globule, pre-molten globule) various types of interactions (attractive and/or repulsive forces) play a significant role [15,22,24,31,32,33]. It has been reported that PEG-protein interaction induced contraction of NalD chains [34]. Na1D is a protein of the TetR family of 212 amino acids, in *Pseudomonas aeruginosa* PAO1, which represses the MexAB-OprM multi-drug efflux operon. The researchers found that NalD interacts with PEG so that individual NalD chains gradually shrink as more PEG chains are added [34]. Other studies have found that macromolecular crowding interactions cause an increase in genome structure and function [35], protein self-association (myoglobin-globular proteins) induced by excluded volume effect [36], and so on.

Since the aqueous phase inside the cell is concentrated with aqueous electrolyte solution and the average crowding of bio-macromolecules (20 to 50% volume fraction), the physicochemical interactions of solutes with water molecules as well as confined bio-macromolecular surfaces and to one another becomes dominant and therefore chemically specific [37]. Hence, all inter-molecular interactions have hard-core repulsion investigated between protein molecules and crowder molecules in the surroundings, favors the folded state of proteins [20,38,39,40,41]. These hard-core repulsions also could result in the compaction of unfolded proteins [42,43,44]. In contrast, the soft interactions, also called chemical interactions demonstrated by various crowders (PEGs, Ficoll, Dextran, and so on), stabilize intermediate states [18,21,22,23,24,31,45]. According to the findings of the prior studies, cellular environments involve a variety of interactions (both repulsive and attractive forces), which result in protein stabilization and/or destabilization. Recently the research article published from our research group showed that the thermodynamic stability of two different proteins (lysozyme and α-lactalbumin) increases when exposed to the mixture of crowding agents (Dextran 40, Dextran 70 and Ficoll 70) at different pH values. Their study showed that the stabilization of proteins increases greater in the presence of mixture system than that of an individual crowder [20]. They also noticed that small crowder molecules appeared to be the governing factor in stabilizing the proteins [20].

Myoglobin (Mb) is a heme-containing globular protein found in abundance in myocyte cells of the heart and skeletal muscle [46]. The protein is abundant in highly oxidative muscle fibers, but its content varies depending on tissue size and form, as well as organisms [47]. Recently polymer crowders and their monomer have been observed to have conflicting effects on the heme-protein (myoglobin, Mb) under similar conditions. Wherein, polyethylene glycol (PEG 10,000 Da) alone led a formation of an intermediate state in the protein and was confirmed to be molten globule (MG) using various spectroscopic techniques [19]. However, a small crowder (PEG 400 Da) had more drastic effects than PEG 10,000 on the protein structure leading formation of a pre-molten globule [22]. Moreover, ethylene glycol (EG) has showed no significant change in the structure of the protein under similar conditions (pH 7.0 and 25 °C), however, it decreases the stability of the protein [38]. These studies confirmed that effect of monomer on the protein structure differs from that of polymers (PEGs) [38].

Here, we compared the effects of individual molecules i.e., PEG 400 and EG, as well as their mixtures (polymer + monomer) at various concentration ratios in mg mL^−1^ (0 + 0, 0 + 50, 0 + 300, 50 + 300, 50 + 0, 300 + 0, 50 + 50 and 300 + 50) on the structure and stability of the protein (Mb). The findings showed that a mixture of crowders leads to an increase in the protein structure and stability compared to that of the individual size of PEG 400. It was also found that the small-size crowder molecule tends to be the governing factor for protein stabilization. Besides this, the study found that exclusion volume leads to protein stability and soft interactions, which lead to structural perturbation and balance of these interactions (attractive and repulsive forces) may be governing factors in cellular conditions required to maintain protein stability and their function.

## 2. Results

### 2.1. Effect of Mixture of Crowders (PEG 400 + EG) on the Structure and Stability of Mb Using Various Spectroscopic Techniques

#### 2.1.1. Influence of PEG 400-EG Mixtures on Absorption Spectra of Mb

**Figure 1:**
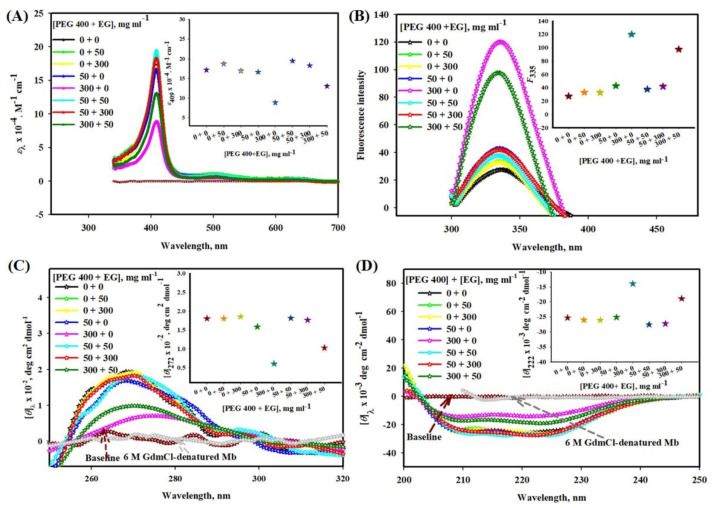
(**A**) Absorption, (**B**) tryptophan fluorescence, (**C**) near-UV CD and (**D**) far-UV CD spectra of Mb with different concentration mixtures of PEG 400 and EG. Insets of (**A**), (**B**), (**C**) and (**D**) displays a plot of ε_409_, *F*_335_, [*θ*]_272_ and [*θ*]_222_ versus various [PEG 400 + EG], where different color stars depicts different [PEG 400 + EG]. All experiments were made at pH 7.0 and 25 °C.

Figure 1A shows the effect of different model systems (PEG 400 + EG) in mg mL^−1^ (milligram per milliliter) on the absorption band (Soret region) of Mb. The inset of the figure depicts a plot absorption coefficient at 409 nm, *ε*_409_ versus various concentration mixture of crowders, [PEG 400 + EG]. The figure suggested that EG does not affect *ε*_409_ while PEG 400 decreases *ε*_409_ at 300 mg mL^−1^. Moreover, the mixture of crowders shows an increase in *ε*_409_.

#### 2.1.2. Influence of PEG 400-EG Mixtures on Mb fluorescence

Figure 1B shows the effect of different model systems (PEG 400 + EG) on fluorescence emission spectra of Mb. The inset of the figure depicts a plot fluorescence emission maxima at 335 nm, *F*_335_ versus [PEG 400 + EG]. The figure suggested that EG does not affect *F*_335_ while PEG 400 increases *F*_335_ at 300 mg mL^−1^. Moreover, the mixture of crowders shows decreases in *F*_335_.

#### 2.1.3. Conformational Studies of Mb in the Presence of PEG 400-EG Mixture and 6 M Guanidinium Chloride (GdmCl) Using Near- and Far-UV CD Spectra

Figure 1C shows the effect of different model systems (PEG 400 + EG) on the near-UV CD of Mb. The inset of the figure depicts a plot of mean residual ellipticity at 272 nm, *θ*_272_ versus [PEG 400 + EG]. The figure suggested that EG has no effect on the near-UV CD spectra of Mb and shows no significant change at 272 nm, while PEG 400 decreases the CD signals, [*θ*]_272_ of Mb completely due to 300 mg mL^−1^ of PEG 400. Moreover, the mixture (PEG 400 + EG) shows an increase in CD signals.

The CD spectrum of Mb in the far-UV region resembles a typical α-helical protein with two significant bands at 208 and 222 nm (see Figure 1D). Figure 1D displays that EG alone does not affect the far-UV CD spectra of Mb. Conversely, PEG 400 alone leads to a decrease in the CD signals of Mb which is maximum due to 300 mg mL^−1^ of PEG 400 [22]. Moreover, their mixture shows an increase in the mean residual ellipticity. Inset of the figure depicts a plot of mean residual ellipticity at 222 nm, *θ*_222_ versus [PEG 400 + EG].

#### 2.1.4. DLS Measurements of Mb in the Absence and Presence of EG, PEG 400 and Their Mixtures

**Figure 2:**
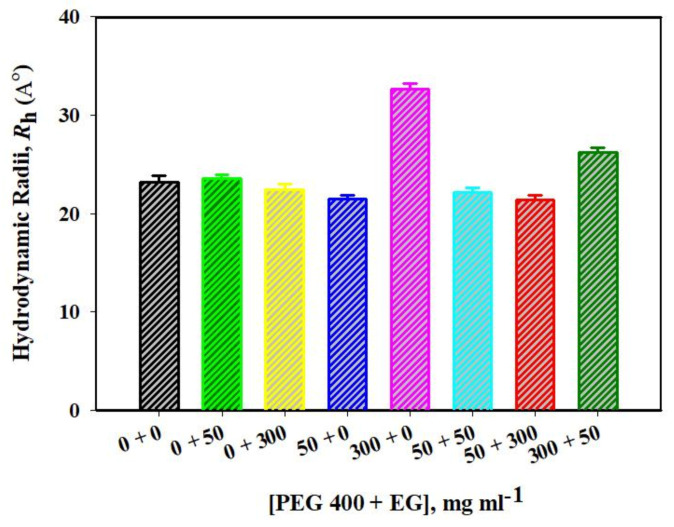
Hydrodynamic radii (in angstrom) of Mb in the presence of different mixture concentrations of PEG 400 and EG analyzed by dynamic light scattering and the different color bars depicts different [PEG 400 + EG].

The hydrodynamic radius (*R*_h_) of Mb in the presence of various [PEG 400 + EG] were estimated using dynamic light scattering (DLS). Each sample measurement gives diameter values in nanometers (nm) using the software Zetasizer Ver. 7.13 of Malvern Panalytical for analysis and the values obtained were converted to *R*_h_ values (see Figure 2). The findings showed that *R*_h_ value of the protein in native condition (Mb alone) obtained is 2.35 nm (23.5 Å) which is close to the value reported earlier [19,22] and that of 6 M GdmCl reported is around 4.9 nm (49.0 Å) [22] which is greater than the protein exposed to mixture (PEG 400 + EG, mg mL^−1^), as shown in the figure.

### 2.2. Binding Studies of Mixture of Crowder Molecules (PEG + EG) with the Protein (Mb)

#### 2.2.1. Isothermal Titration Calorimetry (ITC) Studies

**Figure 3:**
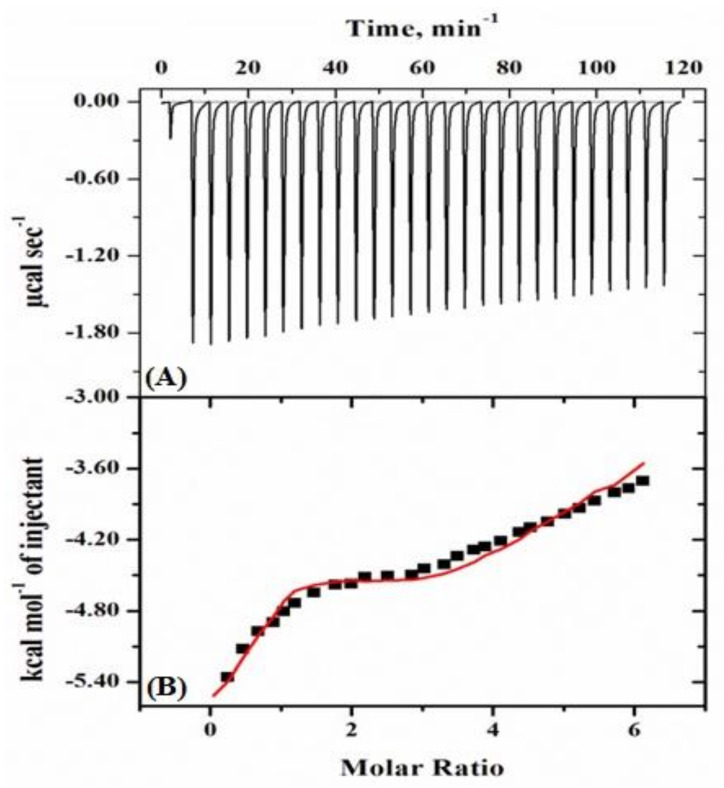
Typical thermogram of Mb with mixture of crowders (PEG 400 + EG) specifies (**A**) calorimetric response to the consecutive injection of the mixture injected to reaction cell and (**B**) provided by subsequent isothermic binding at 7.0 pH and 298 K (25 °C).

Figure 3 displays a typical calorimetric titration profile of a mixture of crowders (PEG 400 + EG) with Mb at pH 7.0 and 298 K (25 °C). In the upper panel (A) of the diagram, each peak in the binding isotherm reflects a single injection of a mixture solution of PEG 400 and EG. The integration of the area under each injection peak in the heat profile gives a differential curve shown in the thermogram’s bottom panel (B) (see Figure 3). Table 1 provides the thermodynamic parameters obtained from the measurements of ITC using sequential binding isotherm.

#### 2.2.2. Molecular Docking Studies

**Figure 4:**
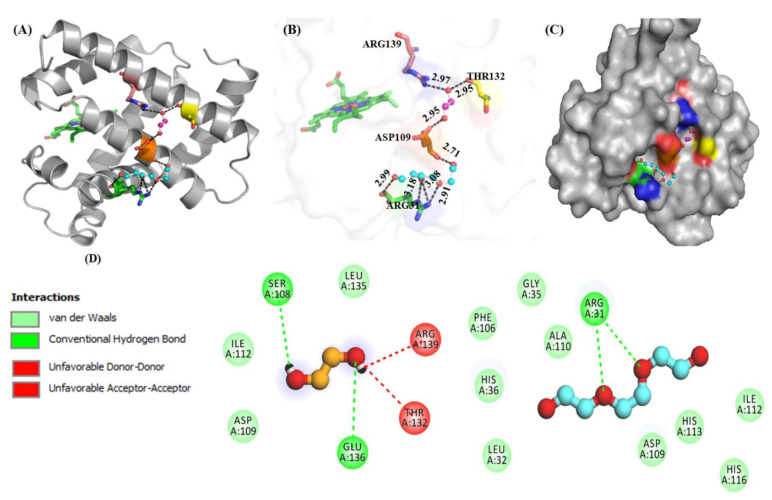
(**A**,**B**) Interactions of crowders (i) PEG 400 (ball and stick model, red-blue) (ii) EG (ball and stick model, red-pink) with amino acid residues of Mb (cartoon model, gray). (**C**) Surface view of Mb and the binding site for PEG and EG on the protein. (**D**) 2D representation of various types of interactions between amino acid residues and crowders (PEG 400 and EG).

Figure 4 shows interaction of molecules (PEG 400 and EG) with residues of Mb through various models. Figure 4A shows the protein (cartoon model)-PEG 400 (ball and stick pattern, blue) and EG (ball and stick pattern, purple) interactions with specific residues. Figure 4B shows the bond distance of crowder molecules (PEG 400 and EG) with specific residues and the bond distances. The surface image of Mb with binding pocket sites for PEG 400 and EG is shown in Figure 4C. Figure 4D represents the 2D structure model of PEG 400 and EG, as well as the protein residues involved in different types of interactions.

**Figure 5:**
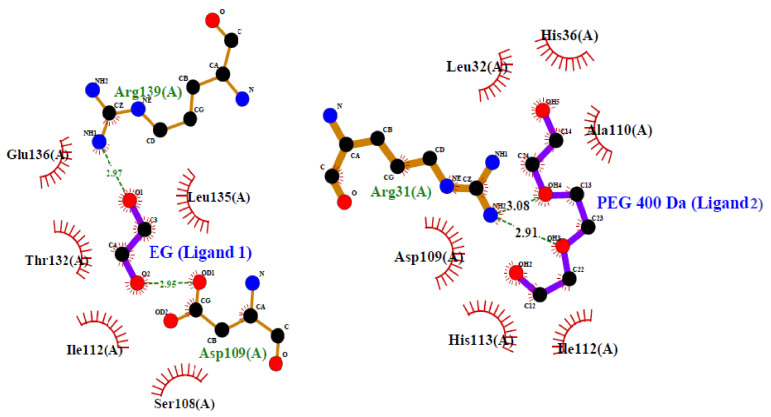
2D-representation of various types of interactions between amino acid residues of Mb with crowders (PEG 400 and EG) using LigPlot.

These various interactions (hydrophobic, H-bonding etc.) between amino acid residues of the protein and crowder molecules (PEG 400 and EG) were further confirmed by using LigPlot (which creates schematic diagrams automatically of protein-ligand interactions for a particular PDB file formed) (see Figure 5).

### 2.3. Heme-Mb Interaction Thermal Stability in the Presence of Various Concentration Mixtures of PEG 400 and EG

It is confirmed from the structural measurements that there is no significant effect due to EG alone on the structure (secondary and tertiary) of Mb. However, PEG 400 alone was seen destabilizing the structure (tertiary and secondary) of the protein (Figure 1A–D). Besides, the findings (see Figure 1A–D) showed that the additives of these crowders showed a slight increase in the structure and decrease in the perturbation of the protein. To see the effect of EG and PEG 400 and their mixtures on the protein stability and heme-globular interactions, thermal denaturation studies were carried out using spectroscopic property, Δ*ε*_409_ versus temperature (*T*).

**Figure 6:**
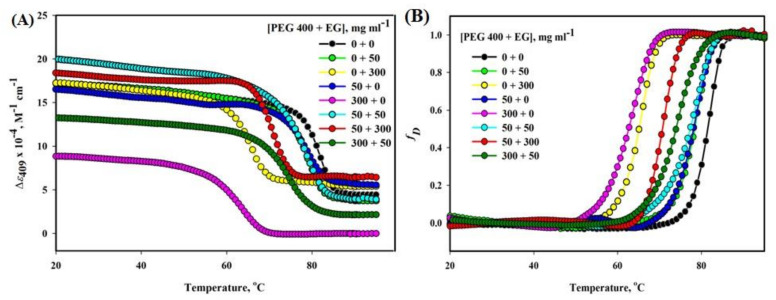
Heat-induced denaturation curves of Mb with different concentration mixtures of PEG 400 + EG, mg mL^−1^ measured using (**A**) probe ε_409_ and (**B**) fraction of denatured protein (*f*_D_) plot.

Thermal denaturation studies of Mb in the presence of different [PEG 400 + EG] were observed at pH 7.0 by using the specific probe Δ*ε*_409_ (see Figure 6). The figure displays that [EG] alone does not affect the temperature-dependence of *y*_N_ (Δ*ε*_409_) but it is affected by [PEG 400] and the mixture of different [PEG 400 + EG]. However, the temperature dependence of *y*_D_ is affected by all the concentrations of each crowder whether alone or together, which is observed to be large at high concentrations. The unfolding curves obtained from UV-visible absorption spectroscopy with parameter values i.e., absorption coefficient at 409 nm (Δ*ε*_409_) of the protein in the presence of crowders and their mixture were plotted in terms of the denatured fraction, *f*_D_ against temperature (see Figure 6B) using linear extrapolation of pre-and post-transition regions and using Equation (4).

The *T*_m_ was observed to be increased in the presence of mixture [PEG 400 + EG] in mg mL^−1^ i.e., 300 + 50 compared to the crowder alone i.e., 300 + 0. Crowders of equal proportion (50 + 50) have a synergic effect (insignificant change in *T*_m_) with that of crowders (EG and PEG 400) alone on the protein. Thermal denaturation curves (Δ*ε*_409_ versus T) of Mb were evaluated at different concentration ratios (PEG 400: EG) using Equation (3) to get the values of *T*_m_ and Δ*H*_m_, provided in Table 2. In the absence and presence of EG, the denatured thermal Mb was >90% reversible, however, in the presence of PEG 400 was completely irreversible and reversible to some extent in the mixture of crowders.

## 3. Discussion

There are numerous studies so far in which proteins are investigated in the individual crowding system with varying crowder concentrations [8,18,19,21,22,23,24,38,39,41,44,48,49,50,51]. However, to comprehend proteins in highly crowded cellular conditions where the pathways of protein folding, structure and stability of proteins are influenced by surrounding biomolecules (macro as well as micro) and how interactions between test protein and biomolecule influences each other, the effect of the mixture of synthetic crowders (PEG 400 and EG) on the structure and stability of the protein (myoglobin, Mb) were investigated using various spectroscopic techniques and binding studies.

The Soret band around 409 nm of Mb (black line) in the visible region (see Figure 1A) has characteristics of a six-coordinated high-spin heme with a histidine residue (His-93) and a water molecule bound at the fifth and the sixth coordination position of the iron atom, correspondingly [52]. Change in the heme environment leads to change in the protein spectrum. The findings suggested that EG alone (at all concentrations) has no noticeable effect in the spectra of the protein and has no effect on heme-globular interactions are unaffected (see Figure 1A). PEG 400, on the other hand, has direct effect on the protein’s heme moiety, causing perturbation at high concentration. These findings were in agreement with those made in previous studies [22,38,53]. Furthermore, the crowder mixture (PEG 400 and EG) did not affect or increased the structure when compared to the te denatured structure of the protein induced by PEG 400 alone (see Figure 1A). The mixture systems [PEG 400 + EG], 50 + 50 and 50 + 300 showed no significant change in the heme milieu and have similar characteristics to native protein (0 + 0 mg mL^−1^). PEG 400 + EG (300 + 0 mg mL^−1^) leads to a decrease in the absorbance, resulting in a change in the heme environment and a disruption of the tertiary structure. When EG (50 mg mL^−1^) and PEG 400 (300 mg mL^−1^) were combined, the results were different, resulting in some protein refolding. All proteins display a characteristic ultraviolet (UV) absorption spectrum around 280 nm due to the aromatic amino acids including tyrosine and tryptophan [53]. This property is used in monitoring the change in the environment of aromatic residues in proteins, hence its tertiary structure [54,55]. EG does not perturb the environment of aromatic residues hence the tertiary structure of Mb at all concentrations [38].

Furthermore, the fluorescence (Figure 1B) and near-UV CD measurements (Figure 1C) were performed to confirm the effects of crowders (alone and combined) on the tertiary structure of the protein (with emphasis on aromatic residues). In native protein, the proximity of the two tryptophan residues (Trp7 and Trp14 on helix A) to the heme moiety results in partial quenching of the tryptophan fluorescence [56]. In the presence of EG, neither the fluorescence intensity of Mb nor shift in *λ*_max_ was observed (see Figure 1B). Similarly, near UV CD measurements (see Figure 1C) revealed that there is no significant change in the Mb’s CD signals at 272 nm, [*θ*]_272_ due to EG, suggesting that the environment of aromatic groups is not perturbed by addition of EG [38]. Conversely, the protein exposed to PEG 400 alone results in an increase in fluorescence emission (small due to 50 mg mL^−1^ and maximum at 300 mg mL^−1^ of PEG 400) (see Figure 1B) and decreases the values of [*θ*]_272_ (small change by 50 mg mL^−1^ and maximum change due to 300 mg mL^−1^ of PEG 400) (see Figure 1C). These findings confirmed that EG does not affect the protein conformation; however, PEG 400 causes the most perturbation in the aromatic group region, which also involves changes in the Trp-heme distances [22]. The model systems (PEG 400 + EG) have a slight or negligible effect on the aromatic environment, as well as a decrease in the protein perturbation due to mixture [300 + 50], mg mL^−1^ (see Figure 1B,C), suggesting protein refolding or/and tertiary structure stabilization of to some extent. The figure insets depicted a plot of different model systems of PEG 400 + EG against *F*_335_ and [*θ*]_272_, which described noticeable changes. It is worth noting that Mb in the presence of polymers of EG (PEG 400 and 10,000) lost most of its tertiary structure at higher concentrations, measured by fluorescence and near-UV CD spectroscopy [22]. Based on the findings above, it can be concluded that EG alone has no effect on the tertiary structure, but when combined with the PEG 400 results either retention or refolding (stabilization) of heme-globular interactions and tertiary structure (heme-Trp distances).

The findings of this study also revealed that EG alone has no significant effect on the secondary structure of Mb, even though half of the secondary structure was perturbed in the presence of PEG 400. The addition of EG (50 mg mL−1) to PEG 400 (300 mg mL−1) in the system (PEG 400 + EG), resulted an increase in the parameters ([*θ*]_λ_) of the protein, resulting in a decrease in the perturbation of protein’s secondary structure (see Figure 1D). Therefore it can be assumed that the mixture of crowders leads to refolding or stabilization of secondary structure to some extent (see Figure 1D). The figure inset depicted a plot of various [PEG 400 + EG] against [*θ*]_222_. Previously, we demonstrated that PEG 10,000 and EG, even at high concentrations, have no effect on CD signals of Mb in the far-UV region [19,38], while PEG 400 has a strong effect at higher concentrations on the secondary structure of the protein [22].

Moreover, dynamic light scattering measurements were carried out (see Figure 2 and Table 3), which showed that there was no significant change in the *R*_h_ values of Mb in the presence of EG alone, and a mixture of crowders (PEG 400 + EG) 50 + 50, 50 + 300. These solutions showed that *R*_h_ values were comparable to each other and close to the native protein (0 + 0 mg mL^−1^). From the above discussion of results, it is confirmed that the protein structure was perturbed in the presence of high concentrations of PEG 400 alone and GdmCl, which increases the size of the denatured protein, resulting in an increase in the *R*_h_ values. The *R*_h_ value of native protein was observed to be 2.35 nm (23.5 Å), as reported earlier [19,22] and that of the protein exposed to PEG 400 without EG (300 + 0 mg mL^−1^) was observed to be 3.24 nm (32.4 Å) and that of 6 M GdmCl exposed was equal to 4.9 nm (49.0 Å) [22]. The values obtained from DLS given in Table 3 suggests that model system [PEG 400 + EG], which includes 300 + 50 mg mL^−1^ leads decrease in the *R*_h_ values of Mb as compared to PEG 400 alone (300 mg mL^−1^), resulting in decrease increase in the size of the protein. These observations showed that mixture of crowders resulted in decrease in the structure perturbation and refolding of the denatured protein. The polymer crowders and their monomer were characterized by their opposing effects on the protein under similar conditions. The crowder molecule, PEG 10,000 was observed to induce molten globule (MG) structure in Mb [19] and PEG 400 was observed to induce pre-molten globule structure of the protein in similar conditions [22]. In addition, EG was observed to have no significant effects on the structure of Mb under similar conditions (pH 7.0 and 25 °C), but decreases thermal stability of the protein with increasing concentration of EG [38].

Table 3 provides the properties of the protein in observed from different spectroscopic techniques in various solvent conditions (buffer, EG, PEG 400 and mixture systems (PEG 400 + EG)). It can be suggested from different parameter values given in the table that EG alone does not affect the structure of the protein, PEG 400 alone induces perturbation at high concentrations and the mixture systems (PEG 400 + EG) for example, 50 + 50 and 50 + 300 mg mL^−1^ has no significant effect on the protein, while the crowded system (300 mg mL^−1^ of PEG 400 + 50 mg mL^−1^ of EG) results in decrease in the perturbation of the protein structure, resulting refolding denatured state of the protein towards the native condition.

Furthermore, the thermal denaturation studies of Mb were observed in the presence of crowders alone and their various mixture ratios ([PEG 400]: [EG]). The crowded system (300 mg mL^−1^ of PEG 400: 50 mg mL^−1^ of EG) showed increase in *T*_m_ of the protein compared to that of crowded individual systems (300 mg mL^−1^ of PEG 400) and (300 mg mL^−1^ of EG). The crowded system with equal proportion of the crowders (50: 50 mg mL^−1^) resulted in an insignificant change in *T*_m_ of the protein compared to that of 50 mg mL^−1^ of both EG and PEG 400 alone (see Figure 6 and Table 2). The figure also showed that the temperature dependence of *y*_N_ (Δ*ε*_409_) is not significantly influenced by [EG] alone, however it depends on the [PEG 400] alone as well as model systems [PEG 400 + EG]. However, the *y*_D_ (temperature dependence) depends on each crowder and at each concentration whether independent or combined and change was greater at higher concentrations. The data analysis confirmed that heme-globular interactions were observed to be recovered and thermally stabilized to some extent in the presence of a mixture of crowders compared to that of individual crowders. The unfolding curves were further processed to denatured fractions, *f*_D_ plots against temperature (see Figure 6B) for better representation and a much easier to understand and measure the effects of co-additives on the temperature midpoint and on the cooperativity (steepness of the curves).

Macromolecular crowding progressively is achieving acceptance in research, i.e., protein folding, therefore showing its nature of action as double-edged sword. The macromolecular crowding shows both destabilizing and stabilizing effects on protein structure, stability and function, depending upon size, shape and concentrations of crowder molecules as well as nature of the protein [15,19,31,38,57,58,59,60,61,62]. A few examples of proteins are examined in the crowded system (mixtures of crowders), where one molecule counteracts another, resulting in structural or/and thermal stabilization [14,20,48,57,58]. Such protein studies in macromolecular crowding represent a big step forward from in vitro to realistically mimicking intracellular conditions. The mixed crowding system exerts a greater stabilizing effect than the sum of the two individual crowding agents [48,57,58]. The recently published research demonstrated that crowded systems (more than one crowder) result in lysozyme stability as their activity declined as the crowder concentration of the mixture increased, as explained by the theory “stability-activity trade-off” [48]. This research also revealed that crowders in the mixture have an adverse impact on the proteins compared to individual crowder molecules [48]. Moreover, conclusions of Shahid et al. [20] concluded that the small size crowder molecule appears to be governing factor for protein stabilization. Our findings also show that the presence of small crowder molecule (here it is EG) in the mixture is governed the structural refolding and increases the thermal stability of the heme protein compared to that of EG and PEG 400 alone.

**Figure 7:**
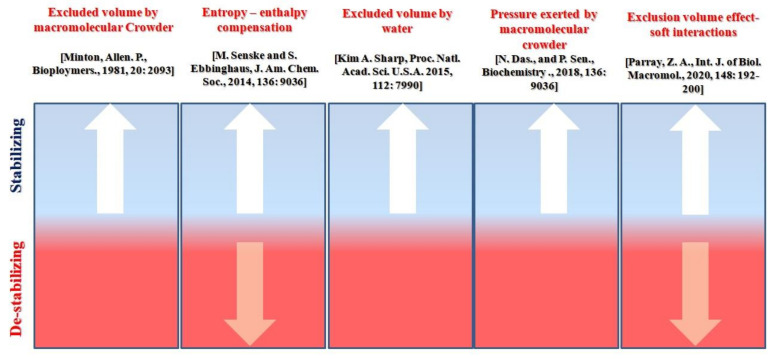
The schematic understanding of protein stability by crowder molecules through various modulations from excluded volume effect alone to excluded volume-soft interactions in a chronological manner.

Recently it was reported [14] that the observed deviation from simple additivity exists at several possible levels or length scales in such mixtures. In addition, the nature and the type of deviation are influenced not only by the identities of the crowder mixture components, but also by the particular surface of the protein where crowders interact. Various kinds of structuring proteins exist in all cells and probably in all organelles, often are peripherally associated with or penetrating through the cytoplasmic inner sheet of the phospholipid bilayer and contributing to the overall mechanical strength, shape and function of a cell or organelle [25]. Our findings may shed light on the possibility of micro heterogeneities in such solutions, which can be mimicked to the cellular solutions in which various molecules interact with one another through repulsive (stabilizing) and attractive (destabilizing) forces interactions. Since we all know cellular conditions are highly crowded, and different mixtures of macromolecules (proteins, DNA, RNA, carbohydrates etc.) interact and affect each other for their subsistence. As a result, two mechanisms of attraction and repulsion may exist in such crowded conditions [2,16,49]. This infers that in medium (in vitro), two mechanisms occur simultaneously, leading to the changes in protein structure and function [24]. Various theories had been proposed which follow the phenomena that configure the macromolecules present in the cytoplasm to perform their biochemical and physiological functions [37]. The negatively charged macromolecules, shape and size of molecules, charges on the surfaces of pathways which acts as switches to regulate cytoplasmic transport of ions and pools having unequal “bulk” concentrations of ionic metabolites are just a few of such theories [37]. As a result, in crowded cellular conditions, the importance of various types of interactions, which play a significant role in protein folding to maintain structure and function, cannot be ignored [15,25,45,63,64]. In vitro crowding assays are now being designed with proteins, which better reflect bio-macromolecular environments under in vivo, allowing for hydrophobic bonding and screened electrostatic interactions [25]. Figure 7 depicts a schematic understanding of protein stability due to crowding agents over time, from excluded volume effect alone to the impact of both excluded volume-soft interactions in a medium [17,22,24,45,63,64,65]. We previously reported that proteins are stabilized by excluded volume (repulsive forces), but proteins are destabilized by soft interactions (attractive forces) [22,24]. From this study, we hypothesize that both the forces are necessary to maintain the structure and function of the protein, and that in cellular conditions, these forces work together to maintain protein-protein and protein-biomolecule interactions, and that intra-intercellular changes (i.e., stabilization-destabilization to maintain equilibrium) result in protein’s function and reliability [2,16].

Furthermore, the thermodynamic binding parameters of Mb with crowder mixture (PEG 400 + EG) were determined using isothermal titration calorimetry (see Figure 3). The binding parameters obtained are given in Table 1. The change in free energy (Δ*G*°) obtained on the interaction of model system (PEG 400 + EG) with Mb is very small (around −3 to −4 kcal mol^−1^). Also, binding affinity (*K*_a_) values are smaller (see Table 1) in comparison to binding studies of the protein with PEG 400 and EG individually [38,39]. The change in binding enthalpy (Δ*H*°) and the change in entropy (Δ*S*°) obtained are highly damaging, resulting in the formation of H-bonds in a low dielectric medium [66].

Moreover, the highly negative value of Δ*H*° denotes that this inter-molecular interaction is exothermic [67]. PEG 400 has previously been shown to bind strongly with the proteins (Mb and cytochrome *c*, cyt *c*) and their heme groups via soft interactions, causing heme-globular interactions to be disrupted [22,24]. Moreover, EG alone showed no specific binding patterns with both the proteins, resulting in a preferential exclusion or/and kosmotropic effect, resulted in no change or slight increase in the structure of the proteins [38,39]. As a result, it is clear that when both the molecules (PEG 400 and EG) bind to the protein at the same time, the soft interactions shown by PEG 400 alone are prevailed by preferential exclusion in the presence of EG, leading the protein towards a stabilizing direction and decreases the protein perturbation. In support of in vitro binding studies, in silico methods (molecular docking) were also exploited to know the binding sites of ligands on the protein, amino acid residues participating in interaction and types of interactions. The molecular docking studies showed (see Figure 4 and Figure 5) that PEG 400 and EG interact with some residues of Mb via weak non-covalent forces without interacting with heme. PEG 400 interacts with Arg31 with bond distances of 2.99, 3.18, 3.08 and 2.91 Å and Asp109 via a single bond of distance 2.71 Å. However, EG interacts with Thr132 and Asp109 with a bond distance of 2.95 Å and Arg139 via a single bond of distance 2.97 Å. Figure 4D depicts the two-dimensional (2D) structure model of ligands interacting with different -protein residues via various types of interactions (conventional H-bonding, van der Waals forces, unfavorable accepter-acceptor and unfavorable donor-donor bonds). These various interactions (hydrophobic, H-bonding etc.) between various residues of Mb with both PEG 400 and EG were further confirmed by using LigPlot (see Figure 5). The computational analysis showed that the interaction of molecules (PEG 400 and EG) with the protein (Mb) gives small binding energies of −3.2 and −2.7 kcal mol^−1^.

## 4. Methodology

### 4.1. Materials

Commercial lyophilized horse heart myoglobin and ethylene glycol (EG) were purchased from Sigma chemical company (St. Louis, MO, USA) and polyethylene glycol of molecular weight 400 Da (PEG 400) from Merck (Mumbai, India). Di-sodium hydrogen phosphate anhydrous and sodium phosphate monobasic anhydrous procured from Himedia (Einhausen, Germany), and all other chemicals utilized were of analytical grade. 0.22 μm pore size filters were purchased from Millipore Corporation (Darmstadt, Germany) and Whatman filter papers from Whatman Laboratories (Cambridge, UK).

### 4.2. Methods

#### Preparation of Solutions of Protein and Reagents

The required amount of lyophilized powdered form of Mb (Cas Number: 100684-32-0) was dissolved in 50 mM phosphate buffer. The solution was then oxidized by potassium ferricyanide [68]. To remove excess of potassium ferricyanide in the solution, the protein solution was dialyzed against several changes of 50 mM phosphate buffer solution at pH 7.0 and 4 °C. After dialysis, the protein solution was filtered through a 0.22 μm Millipore filter and stored at 4 °C for further use. To determine concentrations of Mb, molar absorption coefficient (M^−1^ cm^−1^) value of 171,000 [69] was used. All spectral measurements were taken in triplicates.

The crowder solutions (PEG 400 and EG) and denaturant solution (GdmCl) were prepared in phosphate buffer. Their pH was adjusted to 7.0 using sodium dibasic and monobasic phosphate salts, as required. The crowders and denaturant solutions were filtered through Whatman filter paper no. 1 followed by concentration estimation using refractive index measurements as reported earlier for GdmCl [70] and the crowders [71].

For experimental studies, each protein solution containing the additives (EG, PEG 400 and their mixtures, and GdmCl) was thoroughly mixed and incubated overnight at room temperature, which was sufficient time to attain equilibrium.

### 4.3. Spectroscopic Techniques

#### 4.3.1. Absorption Spectroscopy

Spectral measurements were made in Jasco V-660 UV-vis spectrophotometer (Jasco V-660, Provided by: JASCO Corporation., Ishikawa-machi, Hachioji-shi., Tokyo, Japan) equipped with a programmable Peltier type temperature controller (ETCS761). For absorbance studies (wavelength region of 700–240 nm), 20–25 µM of the protein concentration was used within 1.0 cm path length cuvette. The raw data were converted into molar absorption coefficient using the relation:

*A = εcl* (1)

where *A* is the absorbance, *c* is the molar concentration, *l* is the path length of the cuvette in cm, and *ε* is the molar absorption coefficient (M^−1^ cm^−1^). All spectral measurements were taken in triplicate.

#### 4.3.2. Fluorescence Spectroscopy

Fluorescence spectra were recorded in Jasco FP-6200 Model No.STR-312 Spectrofluorimeter (Provided by: JASCO Corporation., Sennin-cho 2-chome, Hachioji, Tokyo, Japan) at 25 ± 0.1 °C, with both emission and excitation slits fixed at 10 nm. A quartz cell of 1.0 cm path length was used. The cell temperature was controlled with the help of an external thermostated water bath. An amount of 7 µM of the protein was used in all the fluorescence experiments. The excitation wavelength was 280 nm for tryptophan (Trp) fluorescence measurements [56], and emission spectra were recorded in the wavelength region of 300–400 nm.

#### 4.3.3. Circular Dichroism (CD) Spectroscopy

Circular dichroism (CD) measurements were carried out in Jasco Spectropolarimeter, J-1500 model (Provided by: JASCO Corporation., Ishikawa-machi, Hachioji-shi., Tokyo, Japan) attached with a circulatory bath (MCB-100) at 20 °C. Far- and near-UV CD spectra were obtained using protein concentrations of 5–7 µM and 25–29 µM, in 0.1 cm and 1.0 cm path length cuvettes, respectively. The calibration of the machine was consistently done with D-10 camphor sulphonic acid. Each spectrum was corrected for the contribution of the blank. Five scans of each solution were taken to get a better signal-to-noise ratio in all cases together with the baseline. Nitrogen at the rate of 5–6 L min^−1^ was flushed continuously to minimize the noise level. CD data were changed to concentration-independent parameter [*θ*]_λ_ (degcm^2^dmol^−1^), the mean residue ellipticity (MRE), using the relation [72]:

[*θ*]_λ_ = *M*_0_*θ* _λ_/10*lc* (2)

where *θ*_λ_ is ellipticity in millidegrees at wavelength λ, *M*_0_ is the mean residue weight of the protein, *c* is the protein concentration in gm cm^−3^, and *l* is the path length of the cell in centimeter.

#### 4.3.4. Size Distribution Measurements

Malvern Zetasizer Nano ZS instrument (Provided by: Malvern Panalytical, a division of Spectris Co. Ltd. Minato-ku, Tokyo Japan) was used to carry out all size distribution measurements at 25 °C and pH 7.0. The detection angle of 12.8° and the scattering angle of 175° and Helium–Neon laser has a power of 4 mW at the wavelength of 632.8 nm with a beam diameter of 0.63 nm (1/e2) was set in all experiments. The samples of the protein within different solvents conditions, i.e., buffer, EG (50 and 300 mg mL^−1^), PEG 400 (50 and 300 mg mL^−1^) and their various model systems [PEG 400 + EG] were placed in standard Malvern polystyrene cuvettes of 10 mm for size measurements, respectively. The software Zetasizer Ver. 7.13 of Malvern Panalytical was applied for the data analysis. The measurements of each sample were repeated 4–5 times for better results.

#### 4.3.5. Thermal Denaturation Measurements

##### UV-vis Spectrophotometer

Thermal denaturation experiments of Mb were performed in Jasco V-660 UV/Visible Spectrophotometer outfitted with a Peltier-type temperature controller (ETCS-761). The change in the absorbance of the protein with increasing temperature was monitored at 409 nm (to monitor heme-protein interactions and stability). Experiments were performed in the presence of various concentration of mixtures of the crowder (PEG 400 + EG) in mg mL^−1^ (0 + 0, 0 + 50, 0 + 300, 50 + 0, 300 + 0, 50 + 50, 50 + 300 and 300 + 50) at pH 7.0. The protein solution was heated from 20 to 100 °C with a heating rate of 2 °C min^−1^. After denaturation, each protein sample was immediately cooled down to measure the reversibility of the reaction. All solution blanks were subtracted before the analysis of the data [38]. The raw absorbance data was converted into a change in molar absorption coefficient (Δ*ε*_λ_, M^−1^ cm^−1^) at a given wavelength, λ. Each heat-induced transition curve was analyzed for *T*_m_ (midpoint of denaturation) and Δ*H*_m_ (enthalpy change at *T*_m_) using a non-linear least-squares analysis according to the relation [73]:

(3) $$y\left(T\right)=\frac{y_{N}\left(T\right)+y_{D}\left(T\right)\exp[-\Delta H_{m}/R\left(\frac{1}{T-1/T_{m}}\right)}{1+exp[-\Delta H_{m}/R\left(\frac{1}{T-1/T_{m}}\right)}$$

where *y*(*T*) is the optical property at temperature *T* (K), *y*_N_(*T*) and *y*_D_(*T*) are the optical properties of the native and denatured molecules of the protein at temperature *T* (K), and *R* is the gas constant. In the analysis of a denaturation curve, it was assumed that a parabolic function describes the dependence of the optical properties of the native and denatured protein molecules (i.e., *y*_N_(*T*) = *a*_N_ + *b*_N_*T* + *c*_N_*T*^2^, and *y*_D_(*T*) = *a*_D_ + *b*_D_*T* + *c_D_T*^2^, where *a*_N_, *b*_N_, *c*_N_, *a*_D_, *b*_D_, and *c*_D_ are temperature-independent coefficients). The *T*_m_ values obtained were converted from Kelvin (*K*) to degree (°C).

The denaturation data of the unfolding curve obtained by UV absorption spectroscopy were usually plotted in terms of the denatured fraction (*f*_D_) which was calculated from the equation:

(4) $$\;f_{D}=\frac{\left(Y-Y_{N}\right)}{\left(Y_{D}-Y_{N}\right)}$$

where Y is the observed parameter (Δε_409_ at 409 nm) at a given mixture of PEG 400 and EG concentration, Y_N_ and Y_D_ are the values for the native and denatured states of the protein. The values of Y_N_ and Y_D_ were obtained by linear extrapolation of pre-and post-transition regions.

### 4.4. Methods Used for Binding Studies

#### 4.4.1. Isothermal Titration Calorimetry Measurements

VP ITC Calorimeter (MicroCal, Northampton, MA, USA) was employed for isothermal titration calorimetry measurements. The mixture of crowders (PEG 400 + EG) was titrated into a calorimeter cell containing Mb. The protein and crowder mixture ratio was 1:20 and crowder solution (PEG 400 + EG) was prepared with an equal concentration ratio (1:1). The solution (crowders) was filled in a syringe and in every 280 s aliquots of 10 µL were injected except the first injection which was 5 µL. Data were normalized and assessed by software of MicroCal Origin ITC [22,38]. All experiments were carried out in 50 mM phosphate buffer (pH 7.0) at 25 °C (298 K). Origin 8.0 was used to fit the raw data using sequential binding model, which in turn gives thermodynamic parameters including change in enthalpy (Δ*H*°), change in entropy (Δ*S*°) and the association constant (*K*_a_). From these key parameters, change in Gibbs free energy (Δ*G*°) was calculated using equation:

∆G = −R*T*ln*K*_a_ = ∆*H* − *T*∆*S* (5)

where *R* and *T* are gas constant and absolute temperature (in Kelvin) respectively.

#### 4.4.2. Computational Studies (In Silico)

To dock molecules (EG and PEG 400) to a macromolecule (Mb) for with the purpose of virtual molecular screening of compounds, the PyRx software was used [38]. PyRx software is written in Python programming language with an intuitive user interface that run on all major operating systems (Linux, Windows and Mac OS). It is a combination of several softwares, including AutoDockVina, AutoDock 4.2, Mayavi, Open Babel, etc. PyRx uses Vina and AutoDock 4.2 as docking software [74]. The input files of ligand molecules (EG and PEG 400) (Source: Pubchem), and macromolecule, Mb (PDB id: 1ymb) in pdb format were changes to pdbqt files using Autodock software. After preparing the files it was subjected to docking by using AutoDock 4.2 and Vina. Grid box dimensions were set to be X, Y, and Z conformations equal to 50, 42 and 55. The grid space size was assigned perfectly, allowing the search space for the receptor to perform a docking with ligand molecules normally at the binding site. The interaction between Mb with EG and PEG 400 Da was interpreted using the Lamarckian Genetic Algorithm (LGA). Once the Vina calculations were done, results of binding affinity energy (kcal·mol^−1^) of various conformations of the macromolecule with both crowders were provided by the software in a table form. Finally, the best-docked complexes of protein-ligands (EG-Mb-PEG 400) chosen were further modified and analyzed using visualizer PyMOL [75]. BOVIA Discovery Studio, as well as LigPlot v.4.5.3 provided by EMBL-EBI (which uses Java as a programming language) were used to visualize two-dimensional (2D) interaction plots [76,77].

## 5. Conclusions

The study showed that crowders alone and crowders in mixture behave differently with the protein (myoglobin, Mb). Observations showed that EG alone does not affect the structure of the protein, however, it decreased thermal stability of it. Furthermore, the observations showed that PEG 400 alone destabilizes the structure as well as decreases the thermal stability of the protein. In comparison to native protein (0 + 0 mg mL^−1^), a mixture of crowders (PEG 400 + EG) with 50 + 50 and 50 + 300 in mg mL^−1^ showed no substantial improvement in the protein structure, but structural refolding as well as an increase instability of the protein occurs due to mixture ratio [PEG 400: EG] in mg mL^−1^ (300:50). The calorimetric binding studies confirmed that the interaction between the protein and the mixture of crowders is very weak. Previous studies concluded that EG does not affect the protein structure due to preferential exclusion, while PEG 400 alone caused protein perturbation due to soft interactions [22,38]. On the other hand, EG counteracts PEG 400 on the protein structure and stability, resulting in the prevailing of exclusion volume effect over soft interactions, causing the denatured protein to refold. In vitro crowding experiments are now being designed with proteins, which accurately reflect and emphasize bio-macromolecular in vivo conditions. Different proteins stabilize and/or destabilize, allowing various interactions (attractive and repulsive forces) to maintain a high functional protein population.

## Figures and Tables

**Table 1:** Binding parameters of crowder mixture (PEG 400 + EG) with Mb measured from ITC using sequential binding isotherm at pH 7.0 and 298 K (25 °C) ^a^.

Thermodynamic Parameters (Units)	*K*_a_ (M^−1^)	∆*H°* (cal mol^−1^)	∆*S°* (cal mol^−1^ K^−1^)	∆*G°* (cal mol^−1^)
**Step 1**	24,060 (±66)	−22,280 (±649)	−59.2	−4629 (±649)
**Step 2**	996 (±22)	−44,830 (±405)	−137	−3983 (±405)

^a^ A ± with each parameter represents mean error.

**Table 2:** Thermodynamic parameters estimated from thermal denaturation of Mb with various concentration ratio mixtures of PEG 400 + EG in mg mL^−1^ at pH 7.0, using Soret-absorption spectroscopy ^a^.

[PEG 400 +EG], mg mL^−1^	Δ*ε*_409_	
	*T*_m_ (°C)	Δ*H*_m_ (kcal mol^−1^)
0 + 0	81.5 (±0.5)	120.20 (±0.92)
0 + 50	78.6 (±0.60)	120.90 (±0.93)
0 + 300	65.26 (±0.60)	106.30 (±0.82)
50 + 0	78.5 (±0.61)	98.70 (±1.4)
300 + 0	63.2 (±0.60)	70.10 (±0.54)
50 + 50	77.76 (±0.23)	85.02 (±0.70)
50 + 300	70.69 (±0.63)	117.27 (±0.25)
300 + 50	73.75 (±0.5)	90.06 (±0.17)

^a^ A ± with each parameter represents the mean error obtained from triplicate measurement.

**Table 3:** Comparison of spectral properties of Mb in the presence of crowders (PEG 400 and EG) individually and their mixtures (PEG 400+ EG) at pH 7.0 and 25 °C ^a^.

Mixture of Crowders (PEG 400 + EG), mg mL^−1^	*R*_h_ (Å)	*ε*_409_, M^−1^ cm^−1^	*F*_335_	[*θ*] (deg cm^2^ dmol^−1^) at Wavelength in nm	
				222	272
No Crowder (0 + 0)	23.46 (±0.68)	171,645 (±285)	29 (±1.24)	−26,190 (±278)	182 (±12)
(0 + 50)	23.10 (±0.58)	187,716 (±509)	33 (±2.4)	−26,015 (±258)	183 (±14)
(0 + 300)	22.70 (±0.60)	169,831 (±619)	33 (±2.0)	−26,065 (±150)	185 (±10)
(50 + 0)	21.30 (±0.50)	166,561 (±499)	43 (±4.1)	−25,119 (±204)	159 (±7)
(300 + 0)	33.8 (±0.40)	85,776 (±152)	120 (±5.2)	−13,987 (±104)	61 (±4)
(50 + 50)	23.05 (±0.60)	194,821 (±689)	38 (±2.3)	−27,547 (±278)	181 (±12)
(50 + 300)	22.80 (±0.70)	183,029 (±539)	42 (±4.4)	−27,278 (±254)	176 (±10)
(300 + 50)	26.45 (±0.60)	130,644 (±282)	98 (±6.4)	−18,908 (±121)	105 (±6)

^a^ A ± with each parameter represents same as in Table 2.

## Data Availability

The data presented in this study are available on request from the corresponding author.

## Sample Availability

Samples of the compounds are not available from the authors.
